# A policy-driven multifaceted approach for early childhood physical fitness promotion: impacts on body composition and physical fitness in young Chinese children

**DOI:** 10.1186/1471-2431-14-118

**Published:** 2014-05-05

**Authors:** Zhixiong Zhou, Hong Ren, Zenong Yin, Lihong Wang, Kaizhen Wang

**Affiliations:** 1 School Of Physical Education and Coaching Science, Capital University of Physical Education and Sports, Beijing, China; 2 Graduate School of Being Sport University, Beijing, China; 3 School of Sport Sciences, Beijing Sport University, Beijing, China; 4 Department of Health and Kinesiology, University of Texas at San Antonio, San Antonio, Texas, USA; 5 Department of Physical Education Physical Education College of Zhengzhou University, Zhengzhou, China; 6 School of Recreation and Community Sports, Capital University of Physical Education and Sports, Beijing, China

**Keywords:** Multifaceted intervention, Preschool children, Physical activity, Physical fitness, Growth, Body composition, Obesity

## Abstract

**Background:**

The prevalence of obesity increased while certain measures of physical fitness deteriorated in preschool children in China over the past decade. This study tested the effectiveness of a multifaceted intervention that integrated childcare center, families, and community to promote healthy growth and physical fitness in preschool Chinese children.

**Methods:**

This 12-month study was conducted using a quasi-experimental pretest/posttest design with comparison group. The participants were 357 children (mean age = 4.5 year) enrolled in three grade levels in two childcare centers in Beijing, China. The intervention included: 1) childcare center intervention (physical activity policy changes, teacher training, physical education curriculum and food services training), 2) family intervention (parent education, internet website for support, and family events), and 3) community intervention (playground renovation and community health promotion events). The study outcome measures included body composition (percent body fat, fat mass, and muscle mass), Body Mass Index (BMI) and BMI z-score and physical fitness scores in 20-meter agility run (20M-AR), broad jump for distance (BJ), timed 10-jumps, tennis ball throwing (TBT), sit and reach (SR), balance beam walk (BBW), 20-meter crawl (20M-C)), 30-meter sprint (30M-S)) from a norm referenced test. Measures of process evaluation included monitoring of children’s physical activity (activity time and intensity) and food preparation records, and fidelity of intervention protocol implementation.

**Results:**

Children in the intervention center significantly lowered their body fat percent (−1.2%, p < 0.0001), fat mass (−0.55 kg, p <0.0001), and body weight (0.36 kg, p <0.02) and increased muscle mass (0.48 kg, p <0.0001), compared to children in the control center. They also improved all measures of physical fitness except timed 10-jumps (20M-AR: −0.74 seconds, p < 0.0001; BJ: 8.09 cm, p < 0.0001; TBT: 0.52 meters, p < 0.006; SR: 0.88 cm, p < 0.03; BBW: −2.02 seconds, p <0.0001; 30M-S: −0.45 seconds, p < 0.02; 20M-C: −3.36 seconds, p < 0.0001). Process evaluation data showed that the intervention protocol was implemented with high fidelity.

**Conclusions:**

The study demonstrated that a policy-driven multi-faceted intervention can improve preschool children’s body composition and physical fitness. Program efficacy should be tested in a randomized trial.

**Trial registration:**

ChiCTR-ONRC-14004143.

## Background

There is an emerging epidemic of obesity in young children age below 5 years old in both the developed and developing countries [1,2]. Recent national and regional data have consistently shown that the prevalence of obesity in young Chinese children has dramatically increased while fitness measures showed declines since 1980s [3-6]. One study of children in nine large Chinese cities found that the prevalence of obesity in 5 years-old increased from 0.84% in 1986 to 6.05% in 2006, a rate of annual absolute increase at 0.26 percentage point [3]. Recent studies reported prevalence of obesity ranging from 8.4% to 10.5% in preschool children living in large Chinese metropolitan cities [7,8].

According to the 2010 Chinese National Fitness Survey, weight and height of Chinese children aged 3 to 6 years old increased significantly from 2005 to 2010. However, the increase was greatest in weight and Body Mass Index (BMI) and less in height. The survey also revealed that the average National Physical Fitness Index decreased 0.36 percentage points from 2005 to 2010. This trend was also reflected in preschool children with declines in some physical fitness measures (e.g. 20-meter agility run, broad jump, walking on balance beam) [9].

There is an important connection between body growth and physical fitness [10]. Optimal growth is accompanied by healthy body composition characterized by lower level of fat mass and higher level of fat free lean mass (muscles and bone). High level of physical fitness is closely associated with healthy body composition and lower body fat percent in children [11,12]. Participation in physical activities, especially moderate and vigorous physical activity (MVPA), can improve physical fitness and body composition in children [11]. An inverse relationship between levels of obesity and measures of physical fitness was reported recently in a large sample of 6–12 years old Chinese children [13].

Preschool children are surprisingly sedentary and spend more than half of their waking hours being sedentary [14,15]. A recent meta-analysis of 29 studies of preschoolers aged 3–5 conducted in developed countries found that the average of MVPA was 42.8minutes (min)/day(d) [16]. Furthermore, young obese children tend to be less active [17,18] who have lower level of fundamental movement skills [19-21] compared to their normal weight peers. Therefore, effective strategies for promoting MVPA and fundamental movement skills, and reducing sedentary behavior are urgently needed for preschool children [22-24]. There is little data on the amount of physical activity (PA) engaged by preschool Chinese children at the present time. Current regulations of childcare in China require the provision of outdoor play opportunities, play equipment and playground but lack specifics on the amount of activity time and frequency [25].

There is a consensus that multi-component interventions hold the most promise to curtail childhood obesity when both physical activity (PA) and diet are targeted at childcare and/or home [22,26,27]. However, obesity interventions targeted physical activity and physical fitness in children are often preferred since they are relatively straightforward mediators of energy balance and physical growth and pose no known harms [10,28-30]. Dietary interventions are more complex and difficult to implement partly due to the cost of healthier foods and food service regulations [27] and partly due to the fact that children are growing at fast rate and restrictive diet may lead to under-nutrition or malnutrition [31]. This is particular challenging in the developing countries where malnutrition and quality of nutrients in foods are still concerns. Nonetheless, promotion of portion control and intake of fruits and vegetables and reduction of sugar drinks and energy-dense snacks have been linked to success in preventing excessive weight gain, and are recommended strategies for preschool children [32]. Family support and engagement play key roles in developing healthy eating and activity habits in young children. [32,33]. Interventions that targeted both childcare and home have led to more changes in PA and healthy eating, comparing to focusing only on childcare or home [34-36]. Finally, recent reviews have pointed to the need for policy and environmental change studies of early childhood obesity prevention [33,37]. Among the top priorities identified for study are PA policy changes, teacher training in PA, modifications of play format and equipment, engagement of parents, and parent support [27,33,38].

Rapid economic growth in China has led to changes in lifestyle and living conditions, especially in urban regions, in the last few decades [39]. These changes have created an obesogenic environment in large metropolitan regions in China that have reported highest prevalence of obesity in all age groups [4,9]. The correlates of obesity in Chinese children are similar to those reported in studies conducted in other countries, including family background (family income, parent education, parent weight status), birth weight, breastfeeding, physical activity, physical fitness, screen time, diet, access to physical activity and healthy foods, and regions of residence [28,29,40-42]. Obesity preventions targeting these correlates in Chinese children and adolescents have showed promising results [43]. Presently, research is sparse on effective strategies to prevent obesity in preschool children in China. Given the enormity of health and economic consequences of obesity [40,44], there is an urgent need to explore policy and environmental approaches that can address the challenges in combating the obesity epidemic in China. In this paper, we presented the findings of a multifaceted intervention study conducted in childcare centers for preschool children (3–5 years of age) in China. The purposes of the study were 1) to test the effects of the intervention on healthy weight growth (body composition) and physical fitness in preschool Chinese children, and 2) to evaluate the feasibility of conducting a complex health promotion campaign in childcare setting.

## Methods

### Study design and sample

This was a pre- and posttest study with the control group using quasi-experimental design. This 12-month intervention study took place from September 2010 to August 2011. Two public childcare centers in Beijing, China were recruited for inclusion in the study. An important consideration in selecting the intervention center was its proximity to the location of the research team’s institution. Both centers located in inner city area of one municipal district and were 20 kilometers apart. The centers were Class I childcare facilities that met the highest standards of childcare facilities in Beijing and used the same education curriculum [45]. The centers had similar children to teacher ratios, teacher certification requirement (3-year early childhood education), and teacher’s teaching experience. The family income and parental education levels in both centers were also similar. However, the intervention center had higher enrollment, and more indoor and outdoor space.

Children in age range of three to five enrolled in three age-based grade levels. All children were invited to participate in the study. Parents were informed of the study by announcement posters at the beginning of the school year. All parents received consent letters and were asked to provide written consents for their children to participate in the study. No incentive was provided for participation in the study. The study protocol was approved by the Ethics Committee at the Capital University of Physical Education and Sports.

### Description of intervention

#### Theoretical framework in intervention design

The intervention was designed based on social-ecological model (SEM) [45]. and competence motivational theory (CMT) [30]. The SEM stresses multiple leverage points at multiple levels of influences that are important in modifying health behaviors in childcare setting. Following the SEM, the study was designed to target childcare center (policy, teaching training, curriculum, and food preparation), parents (health education and parent engagement), and community (playgroup renovation and community events) in soliciting and supporting systematic changes in children’s physical activity and diet. The CMT was used in design of age-appropriate activity curriculum and play equipment that motivate children to participate in physical activity by increasing their perceived competence, social support and enjoyment of the activities. This was achieved with a games-based approach to movement skills development to enable children to have fun and experience success in developing gross motor skills and physical fitness [46].

#### Intervention design

The multifaceted intervention was created to engage childcare center, families, and community in an integrated effort to promote physical fitness, and support MVPA and healthy eating and to prevent obesity. These objectives were implemented by adopting physical activity and nutrition policy and practices following evidence-based recommendations and by linking physical and health education with health promotion in childcare setting. The intervention had three integral components that were designed to target physical activity and diet behaviors of preschool children using intervention mapping [47]. An overview of the intervention and development and evaluation of intervention components was presented in Additional file 1.

##### Childcare center intervention

The center intervention was designed to change center’s physical activity policy, teacher training, physical education curriculum and food services with full support of the childcare center administrative team.

1. The intervention childcare center adopted a set of policy related to outdoor play time and physical education [48]. Daily required time for outdoor play was 60 minutes (30 minutes in the morning and 30 minutes in the afternoon) for 3-years-old classes and 90 minutes (60 minutes in the morning and 30 minutes in the afternoon) for 4- and 5-years-old classes. In addition, all children took part in a 10-minute exercise routine led by a trained teacher during morning recess. Evaluation standards were also developed for assessing teacher performance in lesson planning and delivery [49].

2. All childcare teachers participated in a 20-hour training (bi-weekly 60-minute sessions) on teaching physical education for preschool children at the beginning of the school year [27]. Topics of training included child growth and development (physical, psychological and gross motor development), design of physical activity and gross motor programs, and pedagogical methods and instructional strategies. They also participated in in-vivo observation and hand-on practices to enhance their confidence in leading the outdoor sessions independently. Attendance rate in teacher training was 100%.

3. A physical education curriculum for outdoor play period was developed based on children’s developmental needs and physical environment at the intervention childcare center [50]. For example, play activities designed for older children were more complex and intense to provide skill challenges and promote physical fitness. Because of limited indoor play space, the play activities were primarily designed for “enclosed” outdoor play ground with concrete surface, while alternative versions of some activities were also created for indoor play during inclement weather. In collaboration with childcare teachers, a five member panel with expertise in pedagogy, child development, and curriculum and instruction designed a curriculum for outdoor-based physical education to promote interest and enjoyment in physical activities and to provide sufficient amount and appropriate types of activities for children of different ages at the center. An exercise routine for the daily 10-minute recess was created incorporated continuous choreographed movements with moderate to vigorous intensity with estimated energy expenditure of 37.06 kcal/kg/min. Trained classroom teachers used the curriculum which included unit plans, and detailed lesson plans, and instructional resources during the outdoor play periods.

4. The implementation of the outdoor physical activity curriculum was closely monitored for quality of the lessons and the amount of physical activity by a nurse practitioner on a daily basis [51]. Led by an expert panel, a monthly class observation of one classroom was conducted to check for quality of instructional delivery (amount and intensity of activities) and to discuss issues and problems encountered during outdoor play periods with the teachers at the intervention childcare center. The feedback was provided to the teachers for improvement.

5. As part of the intervention, the intervention childcare center received child-safe, portable play equipment that was used in implementing the physical education curriculum [52]. The study team designed some of the equipment based on fitness levels and gross motor skill developmental needs of 3–5 years old children. The play equipment was manufactured for the intervention by a local child play equipment manufacture using soft materials to prevent injury. The equipment was portable and assembled quickly with the help from the children. The intervention center also placed drawings of children playing outdoor games and performing different gross motor skills were on the walls surround the outdoor play space and game markings on the outdoor playground and indoor play space. In addition, permanent markings for skipping and hopping games were painted on the ground in both indoor and outdoor play areas. Finally, children were asked to make their own play toys during craft class and to use them during outdoor play.

6. To promote healthy eating and increase the quality of food services, food services workers received two training sessions (3 hours each) by pediatric dietitians [50]. The training included nutrition, food service management for groups, menu design following nutrition standards and regulations for preschool children [53], food preparation and cooking techniques as well as demonstration and hands-on practice of food preparation and cooking techniques for healthy cooking. During intervention, the food service director at the childcare center planed menus to meet the nutrition regulations for childcare and nutrition standards for children [53] and to increase healthy eating choices.

##### Family intervention

The family intervention was designed to formulate a healthy family environment that supported healthy eating and physical activity and discourage sedentary behaviors in children and parents [27,33]. Intervention activities include 1) monthly health education seminars with parents on topics of child physical development, gross motor skill acquisition, family-oriented physical activities, nutrition and healthy food preparation, methods of monitoring and enhancing children’s physical fitness, guidelines for outdoor physical activities, common children’s illness and disease prevention, and promotion of emotional health; 2) 12 monthly newsletters with tips on developing children’s health habits and “Children’s Fitness and Health Handbook” (one for fall and one for spring terms); 3) making of a simple play equipment (bi-monthly) by child and parents that was later used during outdoor play at childcare center; 4) an interactive internet website developed by the study team that provided parents with updates on their child’s changes in physical fitness status and individualized feedback on physical activity and healthy eating and information related physical activity, nutrition and obesity; and 5) family events organized by the childcare center that required the participation of both the child and parents, such as family sports day, family physical activity photograph contest, and family outdoor orienteering.

##### Community intervention

The intervention targeted the neighborhoods surrounding the intervention childcare center and aimed to increase the awareness of childhood obesity and environmental support for physical activity and obesity prevention in collaboration with the neighborhood associations [33]. The intervention included 1) training of the association’s staff and staff designation for child fitness promotion in the neighborhood; 2) renovation of neighborhood child play grounds; 3) installation of child’s play equipment; 4) neighborhood events for promotion of physical activity and fitness in young children; and 5) hosting sports day for families with young children in the neighborhood. Using funding from the study, a 600-square meter playground with soft surface was built in the neighborhood where most of the intervention children resided. Ten large fixed play stations for preschool children were installed. The Community Health Center provided health education to the residents on topics related to physical activity, healthy eating, and prevention of infectious diseases and seasonal illnesses. Two one-day health fairs focused on preschool children were held to provide the residents with health education and counseling by invited experts in child development, nutrition, pediatrics, and physical education. One family sport-day was hosted in the community in Fall that was used to promote the participation in family-oriented physical activity.

### Control condition

Control childcare center implemented an outdoor play program following the childcare standards. Classroom teacher were asked to carry out the outdoor play activities as they normally would and did not receive any training related to obesity prevention and physical activity promotion. There was no change on outdoor play time (60 minutes a day for children in aged 4–5 and 30 minutes a day for 3 years-old) and play activities from the previous year. Food services prepared the meals for the preschool children following the nutrition standards and regulations imposed by the city’s childcare regulatory agency [53]. The food services workers at the control center did not receive any nutrition education and training in meal planning and food preparation. Children and parents in control childcare center did not receive any intervention at home and in their neighborhoods. There was no information exchange among administrators and teachers between the intervention and control center. No intervention was conducted in the communities surrounding the control center. Being 20 kilometers apart also reduced the chance of contamination between the intervention and control community.

### Study measurements

#### Demographic and community information

Parents from both intervention and control centers completed a survey on family demographics (child’s age, gender, and grade level, parental education level and family income) and reported their own height and weight at the beginning of the study. Directors of the Childcare Centers provided information on their staff, curriculum and facilities. Information on communities surrounding the childcare centers was gathered by the research team.

#### Study outcome measures

We used a body composition analyzer (InBody J20, BIOSPACE, Seoul, Korea) that was designed to measure children’s height, weight and body composition with light clothes and without shoes, following the recommended procedure by the manufacturer. The analyzer provided measurements of height, weight, muscle mass, fat mass and percent body fat that have calibrated for infants and preschool children. The analyzer has been shown to have strong validity in young Asian children [54,55]. and used in large intervention trials in children [56]. Body Mass Index (BMI) and BMI z-score for age and gender, and status of overweight and obesity were calculated following the standards recommended by the International Obesity Task Force [57].

We used a battery test from the Chinese National Measurement Standards on People’s Physical Fitness for young children to assess children’s physical fitness, defined as body’s ability to achieve optimal levels of physical performance in dealing with a physiological stress to the body [6]. In adults and adolescents, physical fitness is usually measured by a battery measure against normed references that includes endurance (aerobic fitness), speed, muscle strength, agility, flexibility, body height, and body composition [10]. In young children, physical fitness is assessed by measuring children’s ability in performing fundamental movement skills (gross motor and object manipulative) against age- and gender-normed references underlying the dimensions of physical fitness [6]. Therefore, it is different from criterion-referenced tests of motor skill competence [58] which are commonly used in obesity prevention studies in this age group. Aerobic fitness was usually not measured in this age group in norm referenced tests because of difficulties for young children to follow testing protocol and safety concerns [59]. For example, Fitnessgram, a widely used fitness test battery for school age children in the United States, do not have a test protocol for preschool age children [60]. This normed assessment has been validated in Chinese preschool-age children and used in the Chinese National Fitness Surveys. The measurements included 20-meter agility run for agility and speed, broad jump for leg muscle strength, timed 10-jumps for coordination and leg muscle strength, tennis ball throwing for upper body and abdominal muscle strength, sit and reach for flexibility, balance beam walk for dynamic balance, 20-meter crawl for strength and stamina, and 30-meter sprint. However close to 30% of children (especially young girls) in the study could not complete timed 10-jumps test as required. This measure was not included in data analysis. Standard protocol for the administering the test was followed[6].

The outcome measures were collected at the beginning (September, 2010) and end (August, 2011) of the study by research staff following a standardized measurement protocol. The research staff received training on using the body composition analyzer and administering the fitness test with preschool children and conducted the assessment.

#### Evaluation measures

We conducted extensive process evaluation to assess the feasibility and fidelity of the intervention. The nurse practitioner at the intervention completed daily monitoring report of the outdoor play activities to assure the quality of delivering the play curriculum. To assess differences in levels and patterns of physical activity, a randomly selected group of children from the intervention and control center wore accelerometers (GT3X, ActiGraph Manufacturing Technology Inc., FL., USA)for one week to examine their activity levels and patterns in and outside of childcare during the last month of the intervention [61]. The same group of children also wore heart rate monitors (Polar Team2 Pro, Finland) to assess the activity intensity during outdoor play periods at childcare center. Children attendance (illness-related absence) was also collected to monitor the impact of the intervention on children’s health.

Since the meals were prepared freshly each day by the food service workers in the kitchen at each center, we were able to calculate the amount of foods served to the children per day from food preparation records (the ingredients used in producing the three meals) for 5 weekdays. The data was collected quarterly for a total of 20 weeks (i.e. five consecutive days each quarter) with the assistance of Food Service Director from both the control and intervention centers. The daily average of total energy intake (kcal) and intakes (grams) of fat, carbohydrate, protein, fiber, fruits, and vegetables were estimated using "Chinese Food Nutrients Table” [62] by dividing the total daily amount at each center by total number of children attending on the day.

As part of the process evaluation, parents completed a 60-item Liker-scale health knowledge test on child development, nutrition and physical activity and reported their physical activities (frequency and duration of exercise) at baseline and posttest. Both parents and childcare teachers had their physical fitness assessment based on Chinese Adult Physical Fitness Test Standards [6] at baseline and posttest. Parent attendance in parent health education events at childcare center was also collected. Finally, teachers completed an evaluation survey on the satisfaction and impacts of the teacher training in at posttest.

### Statistical analysis

We used General Linear Models (GMLs) to test the differences in change scores of the outcome measures from baseline to posttest between the intervention and control centers. Child’s gender, grade level in childcare, pre-test measure, parent education levels, family income, and parental obesity were included in the model as covariates. We also tested interactions between treatment condition, child’s gender, and grade level in childcare. Only signficant terms were retained in the model. Estimated differences of mean changes and their 95% confidnce intervals were provided. Chi-square tests were used to compare changes in levels of participantion in physical activity and physical fitness from baseline to posttest from parental and childcare teacher surveys in the intervention center. Independent-samples t-test was used to test the differences in energy expenditure at center and at home and heart rates during outdoor play between the intervention and control centers at the end of the school year, and the average daily energy intake and intakes of fat, carbohydrate, protein, fiber, fruits and vegetables. The difference in parent health knowledge test scores between two treatment conditions was tested with GLM controlling baseline scores. The signifiance of all tests were set at p < .05 (two-tailed test). IBM SPSS Statistics (version 18) was used for data analysis.

## Results

### Characteristics of study sample

**Figure 1:**
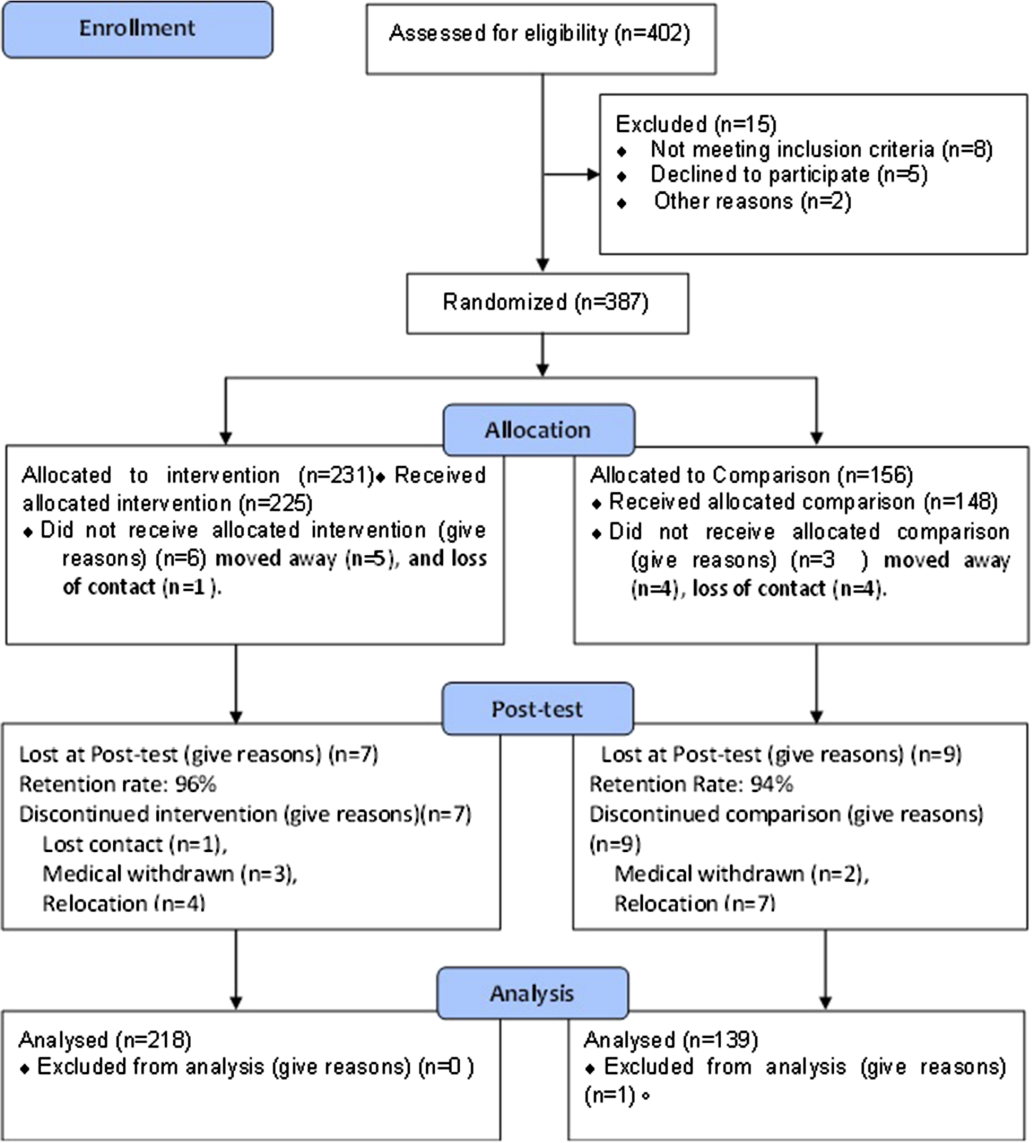
Study participant flow diagram.

We obtained parent consent from 387 children to participate in the study. The participation rate was 96.2%. Three hundred and fifty-seven children were retested at posttest with a retention rate of 95.7%. Figure 1 shows the flow of the study participants. Data analysis was performed on children with both baseline and posttest weight (N = 357). There were more children in intervention center than the control center across three grades. The characteristics of the study sample is shown in Additional file 2. Family monthly incomes were significantly higher in control children. Fathers of control children were more likely to be overweight and obese compared to fathers of intervention children.

### Baseline treatment equivalence check

To assure treatment equivalence at baseline, we examined the outcome measures by children’s grade levels in childcare and gender between intervention and control conditions at the baseline. The results are presented in Table 1. There were significant trends that the anthropometric and body composition measures increased and fitness measures improved with grade levels in childcare. Muscle mass was higher in third year boys than girls. Boys had better performances on tennis ball throw, 30-m craw, and flexibility measures than girls across all grades. No other grade level or gender difference was found. There was no significant interaction effect of years in childcare and gender on the outcome measures. We also did not found difference on the outcome measures at baseline between children who returned and those who did not return for posttest. We found no significant difference between children who returned for posttest and those who did not on outcome measures and family characteristics (data not shown).

**Table 1:** Comparisons of study outcome measures at baseline test (N = 357) ł

	**All**		**First grade (n = 116)**				**Second grade (n = 131)**				**Third grade (n = 110)**			
			**Boys (n = 61)**		**Girls (n = 55)**		**Boys (71)**		**Girls (60)**		**Boys (n = 59)**		**Girls (n = 51)**	
	**Mean**	**SD**	**Mean**	**SD**	**Mean**	**SD**	**Mean**	**SD**	**Mean**	**SD**	**Mean**	**SD**	**Mean**	**SD**
Body fat percent†	21.17	4.46	19.82	4.56	21.54	4.07	20.10	3.39	21.20	4.52	22.87	4.56	21.89	5.16
Fat mass (kg)	3.64	1.25	3.28	0.88	3.49	0.94	3.28	0.92	3.71	1.08	4.20	1.86	4.00	1.36
Muscle mass (kg) † ˥	6.72	1.39	5.84	1.03	5.75	1.04	6.60	1.15	6.50	1.04	8.22	1.34	7.50	0.84
Weight (kg) †	18.16	2.95	16.40	2.13	16.47	1.98	17.65	2.34	17.95	2.38	21.03	3.46	19.75	2.08
Height (cm) †	107.28	6.73	101.56	4.35	100.72	4.53	107.13	4.40	107.12	4.40	114.70	4.26	113.02	4.51
BMI‡	15.71	1.41	15.84	1.18	16.21	1.38	15.33	1.15	15.60	1.39	15.91	1.87	15.45	1.28
BMI-for-age z-score ‡	0.23	0.96	0.27	0.89	0.57	0.90	0.01	0.85	0.17	0.88	0.35	1.27	0.07	0.82
20m agility run (seconds) ‡	8.39	1.63	9.74	1.77	9.91	1.65	7.77	0.88	8.20	1.04	7.25	0.94	7.55	1.13
Broad jump (m) †	90.78	16.03	76.67	7.03	74.80	7.36	90.67	12.54	88.75	15.01	104.71	15.10	99.76	11.81
Tennis ball throw (m) † ˥	4.33	1.68	3.64	1.44	2.98	0.97	4.35	1.28	3.83	1.38	6.34	1.58	4.87	1.11
Sit and reach (cm) ‡ ˥	9.94	4.23	10.11	4.01	10.62	3.60	8.98	3.56	11.09	3.60	7.70	4.76	11.63	4.75
Balance beam walk (seconds) ‡	14.43	12.49	22.40	13.39	24.83	16.77	12.38	9.31	11.72	7.75	7.76	7.58	7.43	4.36
20m craw (seconds) ‡ ˥	26.17	6.38	30.32	5.35	32.09	5.44	24.54	4.01	26.97	6.52	20.71	4.15	22.50	4.24
30m sprint (seconds) ‡	9.84	2.33	11.47	2.61	11.92	2.65	9.46	1.59	9.77	1.59	8.17	1.04	8.18	0.86

ł Comparison based on F-test (α ≤ 0.05); sample size varied slightly for different measures due to missing. † Measure significantly increased with years in childcare; ‡ Measures significantly decreased with years in childcare; ˥ Measures significantly better in boys;

### Intervention effects on study outcomes

Results of regression analysis on the outcome measures are presented in Table 2. There were significant increases in children’s weight (0.36 kg, p <0.02), height (0.47cm, p <0.01,), and muscle mass (0.48 kg, p <0.0001) in intervention children compared to control children. Children in the intervention center also had significant decreases in percent body fat (−1.2%, p < 0.0001) and fat mass (−0.55 kg, p <0.0001) than control children. Children in the intervention center had significant favorable increases in 20-meter agility run (−0.74 seconds), broad jump for distance(8.09 cm, p < 0.0001), tennis ball throwing (0.52 meters, p < 0.006), sit and reach (0.88 cm, p < 0.03), balance beam walk (−2.02 seconds, p <0.0001), 30-meter sprint (−0.45 seconds, p < 0.02), and 20-meter crawl (−3.36 seconds, p <0.0001), compared to children in the control center. Baseline measure of the outcome, father’s BMI, and grade levels influenced the variations in most of the outcomes. There was no significant intervention effect of treatment condition and child’s grade levels in childcare or treatment and child’s gender on the outcome measures.

**Table 2:** Means and Standard Errors (SE) at baseline and posttest and comparisons of intervention impacts on study outcome measures (N = 357)

	**Control (n = 139)**				**Intervention (n = 218)**				**Adjust difference of mean change (95% CI)ł**	**α ≤**
	**Baseline**		**Posttest**		**Baseline**		**Posttest**			
	**Mean**	**SE**	**Mean**	**SE**	**Mean**	**SE**	**Mean**	**SE**		
Weight (kg)	17.77	0.22	19.52	0.25	18.42	0.21	20.53	0.22	0.36 (0.06, 0.66)	0.02
Height (cm)	106.47	0.55	111.12	0.56	107.80	0.46	112.92	0.47	0.47 (0.19, 0.75)	0.01
Body Mass Index (BMI, kg/m^2^)	15.64	0.12	15.78	0.14	15.76	0.09	16.04	0.10	0.19 (−0.06, 0.43)	n.s.
BMI-for-age z-score	0.19	0.08	0.26	0.09	0.26	0.07	0.44	0.07	0.15 (−0.01, 0.31)	n.s.
Body fat percent ł	20.64	0.37	21.45	0.37	21.51	0.30	20.79	0.28	−1.2 (−1.85, −0.79)	0.0001
Fat mass (kg) ł	3.51	0.08	4.25	0.12	3.72	0.09	3.84	0.10	−0.55 (−0.72, −0.39)	0.0001
Muscle mass (kg)	6.59	0.11	7.29	0.13	6.80	0.10	7.96	0.11	0.48 (0.33, 0.62)	0.0001
20m agility run (seconds) ł†	8.56	0.15	7.97	0.14	8.28	0.10	7.06	0.06	−0.74 (−0.89, −0.58)	0.0001
Broad jump (cm) ł†‡	89.35	1.36	99.75	1.20	91.76	1.19	110.05	1.14	8.09 (6.24, 9.93)	0.0001
Tennis ball throw (m) ł†±	4.39	0.14	4.81	0.17	4.30	0.11	.39	0.13	0.52 (0.15, 0.88)	0.006
Sit-and-reach (cm) ł	10.65	0.31	10.99	0.35	9.49	0.31	11.32	0.27	0.88 (0.10, 1.66)	0.03
Balance beam walk (seconds) ł†	14.65	1.16	7.74	0.59	14.29	0.79	5.48	0.27	−2.02 (−3.05, −0.99)	0.0001
20m craw (seconds) ł	25.74	0.52	22.92	0.46	26.45	0.44	20.04	0.34	−3.36 (−4.02, −2.69)	0.0001
30m sprint (seconds) ł†‡	10.09	0.22	9.03	0.17	9.68	0.14	8.14	0.08	−0.45 (−0.82, −0.08)	0.02

ł Adjusted for baseline measure;†Adjusted for grade level;‡Adjusted for father’s BMI;± Adjusted for father’s education.

### Evaluation of Intervention Process Measures

Overall, the intervention was delivered with high fidelity. Based on monitoring reports from the head nurse at the intervention center and observations of study staff that with a few exceptions, teachers in the intervention center conducted the outdoor play (weather permitting) using the weekly unit plan and daily lesson plans developed by the study team on daily basis. Increased time allotment for PA (60-min for 3-years old and 90-min for 4- and 5-years old) was followed throughout the year. We also examined the levels of activity intensity by heart rate monitor during outdoor play and energy expenditures at and outside the center from a group of children (see Table 3). Compared to children in the control center, intervention children had higher heart rates during outdoor play, total daily activity expenditure and energy expenditure and amount of time from MVPA at center. MVPA expenditure and minutes in intervention children outside center during weekdays was higher than control children; but no difference on weekend days. Based on the analysis of food preparation records, the children in the intervention center were served meals with reduced fat (−9.76 g, p > .05), increased fiber (+2.69 g, p < .04) and fruits (+43 g, p > .0001), compared to the children in the control center. There was no difference in daily total energy intake, and intakes of carbohydrate, protein, and vegetables in meals served between the intervention and control centers. Finally, monitoring of children’s attendance showed that absences due to illness remained low and changed from 5.1% to 2.5% in the control center and from 5.9% to 1.5% in the intervention center.

**Table 3:** Comparisons of heart rates and energy expenditures of control and intervention children at posttest

	**Control**	**Intervention**
Average heart rates (bpm/min) during outdoor play at center †	120 (SD 17; range 78–169)	128 (SD 18; range 85–181)
Daily moderate and vigorous physical activity expenditure at center (Kcal) ‡	152.00 (SD 42.11)	242.08 (SD 73.40)
Daily moderate and vigorous physical activity expenditure outside childcare (Kcal) ‡	137.56 (SD 2.98)	196.11 (SD 4.26)
Daily moderate and vigorous physical activity minutes at center (min)‡	30.91 (SD 8.99)	59.76 (SD 10.54)
Daily moderate and vigorous physical activity minutes outside center (min)‡	21.44(SD 4.75)	35.67(SD6.41)
Daily moderate and vigorous physical activity minutes on weekend days (min)‡	50.03(SD18.02)	68.97 (SD19.57)

Children attended childcare from 8 am to 5 pm including lunch and a noontime nap. † α ≤ 0.01; ‡ α ≤ 0.0001.

Teacher had positive responses to the teacher training and had 100% attendance in all training sessions. In the post study survey, teachers reported improvement in the following areas as result of the training and continued monitoring and evaluation: understanding of the importance of physical education (76.6%), increased ability in curriculum development (62.7%), organization and management of outdoor play (80.1%), increased ability in researching curricular issues (56%), understanding of movement and skill development in preschool children (100%), understanding of the goals and objectives of preschool physical education/outdoor play (98.5%), design of age-appropriate physical activities (100%), creating a safe play environment (92.7%), setting up the equipment and fields for physical activities (90.6%), selection and use of teaching methods and strategies (94.4%), controlling activity load and intensity (100%), motivating children (89.7%), monitoring, providing feedback and making adjustment in class activities (98.4%), developing disciplines (92.5%), and communicating and coordinating teaching activities with colleagues (95.7%).

There was a high level of parent engagement in intervention activities. The average rate of parent attendance in the four parent education events was 94% (92% in 3-years old, 95% in 4-years old, and 96% in 5-years old). Intervention parents significantly improved their scores on the health knowledge test (p ≤ 0.05) from 25 at baseline to 51 at posttest, compared to control parents (from 26 to 31). Results of parent’s report of exercise and fitness levels based fitness assessment showed positive changes from baseline to posttest (see Additional file 3). Intervention parents increased frequency and duration of exercise from baseline to posttest. Levels of fitness also increased in parents and teachers in the intervention center from baseline to posttest. Ninety-two percent of the parents reported that the internet website provided them useful information to manage child’s health and promote physical activity and healthy eating.

## Discussion

Findings from this study demonstrated that a multi-faceted intervention can improve preschool children’s body composition and physical fitness. Children in the intervention center had less gains in body fat percent (−1.35%) and fat mass (−0.55 kg) and more gain in muscle mass (+0.48 kg) and total body weight (+0.36 kg) than the children in the control center. The additional gain in body weight (+0.43 kg) may be attributed to the increase in bone content in the intervention children although it was not assessed in the study. However, it should be noted that there was no significant differences in BMI and BMI z-scores between intervention and control children. As all preschool children are expected to grow taller and heavier, the intervention clearly promoted the development of fat free soft tissues and prevented excessive weight gain. This is similar to the results of a physical activity intervention study that significantly reduced body fat percent, and increased fat free mass and bone density but increased body weight and BMI in elementary school children [63,64]. The body composition in that study was assessed by Dual-energy X-ray absorptiometry. Both study used a robust physical activity intervention that may account for “the healthy” weight gain in intervention children with more fat free soft tissues and less fat mass [31]. The present study added 28 minutes of MVPA in intervention children on weekdays and 18 minutes of MVPA on weekend days. A Swedish study also reported an increase in physical fitness and decrease in skinfolds in elementary school children in a 1-year physical activity intervention study [56]. The Framingham Children's Study tracked physical activity by accelerometry and body fat by triceps and subscapular skinfolds in preschool children aged 3 to 5 years from preschool to the first grade in school and found that active preschool children gained significantly less fat compared to inactive children after controlling television viewing, energy intake, baseline triceps, and parents' body mass index [65]. The 2011 Cochrane review on childhood obesity prevention reported an average reduction of 0.26 kg/m^2^ in children aged 0–5 years from 5 RCTs, conducted mostly in the developed countries [26]. The impact on BMI appeared to be the strongest in the youngest children than those aged 6–12 years and 13–18 [26]. A recently completed community-based multi-setting and multi-strategy obesity prevention intervention significantly lowered weight, BMI, BMI z-score, and prevalence of overweight/obesity in subsamples of 2 to 3.5 years old Australian children [66]. The finding of impact on body composition from this study adds to the literature on this important topic.

The intervention significantly increased the performances in 20-meter agility run, broad jump, tennis ball throwing, sit and reach, balance beam walk, and 20-meter crawl in intervention children. These measures are manifestations of children’s fundamental movement skills and movement capabilities [6]. Few obesity prevention studies measured their impacts on physical fitness in young children [67]. A large randomized trial with Scottish preschool children found that a physical activity intervention significantly improved fundamental movement skills but had no impact on weight and BMI [68]. The authors attributed the lack of effects on weight and BMI to inadequate dose of physical activity. Significant improvement in fundamental movement skills was also reported in low-income preschool children who were exposed to gross motor activity interventions in the United States [34,38]. The improvement in physical fitness measures in this study was attributed to the provision of physical activity programs (70–100 minutes daily at the center) that incorporated age-appropriate fundamental movement skills and gross motor activities with moderate and vigorous intensity [69-71]. The quality of outdoor play was further enhanced by innovative, soft and portable playgroup equipment designed specifically to meet the developmental needs of fundamental movement skills for preschool children [51,52]. Similar findings have also been reported in older children who were exposed to MVPA intervention [11,72].

The results of the process evaluation suggested that the policy and environmental changes at childcare center may account for the enhancement of teacher’s ability in implementing the outdoor curriculum increased the amount of MVPA, nutritional quality of food services, produced supportive environment for physical activity and healthy eating in preschool children. These changes have been identified as effective strategies for childhood obesity prevention by the recent Cochrane review [26] and others [33,38]. Policy and environmental interventions in childcare setting in the United States [73] and Australia [74,75] have led to changes in children’s play behaviors, increase in structured play time, and improvement in teacher training. Intervention with parents was successful and well received as indicated by high level of participation. The family intervention increased parental support and engagement [76,77]. and modeling [78] that encouraged children to be more active at home. The environmental changes, such as playground renovation and health promotion events in intervention community can contribute to increased access to physical activity opportunities and health education in the community [33], although we did not collect information on the use and attendance of study participants to affirm the potential impact of the community-based intervention.

The study has several strengths that increased the internal validity of the study. First, we received full cooperation and participation from the center administration and staff to implement our proposed policy and environmental changes which provided unique opportunity to test their impacts. High fidelity of implementation was the results of this support and cooperation. Secondly, the uses of validated age-appropriate outcome measures were critical to accurately assess the impacts of the study in young study participants. For example, without the use of the bioimpedance analyzer, we will not be able to observe the favorable changes of body composition, i.e. decreased body fat percent and increased muscle mass, in the intervention children [79]. Third, we took consideration of children’s age, developmental needs and environmental barriers in designing intervention activities [26,27]. As a result, all children benefited similarly, regardless their gender and grade level.

There were limitations in the study design that warrant cautions in interpreting the study findings. The study had a small sample size and used a non-randomized study design with two conveniently selected childcare centers. We tried to minimize the threat of selection bias to the study validity by using a control center that matched the intervention center in demographics and quality of the facilities and teachers. Compared to the physical activity intervention, we had a modest intensity nutrition intervention with food service workers and parents and did not have direct nutrition education to the children. Nutrition education with children can increased healthy eating behaviors in preschool children and should be included in future [27]. We only collected limited dietary data (food preparation records) which hindered the understanding of the impact on dietary behaviors. No valid dietary measure was available for preschool age Chinese children [80]. Changes in parental physical activity were based on self-report and need to be validated in the future. Finally, we did not conduct long-term follow-up to examine the sustainability of the study impacts on the children and the facility.

## Conclusions

We conclude that the policy-driven multi-faceted intervention, that were designed to target physical activity and diet behaviors of preschool children, significantly improved preschool children’s body composition and physical fitness. The design of the intervention has taken many of the research priorities identified in the literature. To our knowledge, this was the first such study in China. The intervention program was effective to engage children, childcare center staff, families, and community in an integrated effort to promote physical fitness and healthy eating by link policy and environmental changes, physical and health education and health promotion in childcare setting. Findings from this study may contribute to the understanding of intervention design and curriculum development in early childhood obesity prevention in China and other countries in similar stages of economic development and societal changes [30]. Although it is not expected that our intervention program can be adopted in whole by any community or school system, the findings from this study can be informative for health professionals and researchers in formulating intervention in early childhood obesity prevention. We plan to make the outdoor play curriculum and intervention materials designed for the study available for others to replicate the study. The intervention program should be tested for efficacy as well as cost-effectiveness in a randomized trial.

## Competing interests

The authors declare that they have no competing interests.

## Authors’ contributions

ZZ was responsible for study design, study protocol development and implementation, data collection, and preparation of the manuscript; RH participated in study design, study protocol development, data collection, interpretation of data analysis results, and preparation of the manuscript; YZ was responsible for study design,data quality check, data analysis, and preparation of the manuscript; WL was responsible for study protocol implementation, data collection; WK was responsible for securing the funding for the study, conceptualization and design of the study, supervision of the study protocol implementation, and drafting the manuscript. All authors read and approved the final manuscript.

## Pre-publication history

The pre-publication history for this paper can be accessed here:

http://www.biomedcentral.com/1471-2431/14/118/prepub

## Supplementary Material

Additional file 1 Components of Intervention. Click here for file

Additional file 2 Study participants’ characteristics. Click here for file

Additional file 3 Changes of reported exercise and fitness assessment in intervention parents and teachers from baseline to posttest. Click here for file
