# Supplementary material for: A policy-driven multifaceted approach for early childhood physical fitness promotion: impacts on body composition and physical fitness in young Chinese children
Source: BMC Pediatr. 2014 May 5;14:118. doi: 10.1186/1471-2431-14-118 (PMC4108008; doi:10.1186/1471-2431-14-118)
Supplement: Additional file 1 — Components of Intervention. [file 1471-2431-14-118-S1.pdf]

Additional file 1 –Components of Intervention

	Center-based Intervention			Home-based Intervention	Community-based Intervention
	Physical activity Program	Staff Development	Food Service		
Objectives	• Providing 60-90 min of structured and free play<br>• Increasing intensity level<br>• Developing physical fitness	• Improving staff knowledge and skills in physical activity<br>• Enhance staff confidence in leading the outdoor sessions<br>• Improving staff wellness	• To promote healthy eating<br>• Increase the quality of food services	• Formulating healthy family environment for healthy eating and physical activity<br>• Increasing health literacy on obesity, nutrition and physical activity	• Increasing the awareness of childhood obesity and environmental support
Intervention activities	• Provision of gross motor activity equipment<br>• Designed physical education curriculum for outdoor play<br>• Daily 10-minute recess<br>• Monitored outdoor physical activity curriculum<br>• Designed and provided portable play equipment<br>• Placed drawings of playing outdoor games<br>• Children making their play toys	• 20-hour training sessions included child growth and development, design of physical activity and gross motor programs, pedagogical methods and instructional strategies<br>• In-vivo observation and hand-on practices<br>• Staff health screenings<br>• Staff nutrition education	• Two training sessions included nutrition, food service management for groups, menu design following nutrition standards and regulations<br>• Demonstration and hands-on practice of food preparation and cooking techniques	• Monthly health education seminars<br>• 12 newsletters for family members<br>• 2 Children’s Fitness and Health Handbook<br>• 3 family events organized<br>• Making of a simple play equipment (bi-monthly) with children<br>• Interactive discussion on specialized internet website	• Training of the staff designation in the neighborhood<br>• Building a 600-square meter playground<br>• Ten large fixed play stations<br>• Provided health education<br>• Neighborhood events<br>• Hosting sports day for families<br>• 2 one-day health fairs<br>• 1 family sport-day
Development of intervention activities	• Research team and invited experts<br>• Based on the Guidelines for 3-6 Years Old Children's Learning and Development in China and the Curricular Guidelines for Preschool Education	• Research team and invited experts<br>• Based on the Nursery Teacher Professional Development Standards	• Nutrition experts<br>• Based on the Chinese Children's Nutrition and Feeding Guidelines and Children's Meals Management Routines in Childcare Centers	• Research team and invited experts<br>• Based on the Guidelines for Family Engagement in Early Childhood Education	• Research team and community health center<br>• Based on People's Republic of China Sports Law and the Recommendations on Strengthening the Work of Urban Community Sports
Delivery of intervention	• Childcare center stafft<br>• Research assistants<br>• Nurse practitioner<br>• Children and his/her parents	• Expert panel<br>• Childcare center staff<br>• Research staff	• Pediatric dietitians<br>• Peer services workers	• Expert panel<br>• Research staff<br>• Childcare center staff	• Community Health Center<br>• Research staff<br>• Childcare center staff
Training provided	• Use of activity cards<br>• Leading outdoor play activities<br>• Gross motor development	• Workshops on training and hand-on practices<br>• Counseling on physical	• Workshops on training<br>• Presentation skills<br>• Counseling on nutrition	• Delivery of session materials<br>• Presentation skills	• Workshops on training<br>• Installation equipment<br>• Organized family events

		activity and healthy eating	regulations and standards	• Developed interactive internet website	
Evaluation	• Energy expenditure and minutes of MVPA<br>• Daily activity expenditure<br>• Activity intensity by heart rate<br>• Children attendance (illness-related absence)	• Attendance in training sessions<br>• Evaluation survey on the satisfaction and impacts of the teacher training<br>• Physical fitness assessment	• Average of total energy intake<br>• Intakes of carbohydrate, protein, and vegetables	• Parent engagement in intervention activities<br>• 60-item Liker-scale health knowledge test<br>• Reported exercise<br>• Fitness assessment	• The number of play equipment renovated<br>• Footage of playground area renovated<br>• Number of events hosted in the intervention community
