# Supplementary material for: A policy-driven multifaceted approach for early childhood physical fitness promotion: impacts on body composition and physical fitness in young Chinese children
Source: BMC Pediatr. 2014 May 5;14:118. doi: 10.1186/1471-2431-14-118 (PMC4108008; doi:10.1186/1471-2431-14-118)
Supplement: Additional file 2 — Study participants’ characteristics. [file 1471-2431-14-118-S2.pdf]

Additional file 2 –Study participants' characteristics

		Treatment Group		Group comparison†
		Control (C; n=139)	Treatment (I; n=218)	
Grade level	First grade	48	68	
	Second grade	55	76	
	Third grade	36	74	I>C
Children age in months		52.74/9.12	54.10/9.50	n.s.
Family monthly income (RMB)		1,1165/2155	1,0290/3407	C>I
Father education	<=High school	2%	8%	
	College/university	64%	58%	
	Postgraduate	34%	34%	n.s
Mother education	<=High school	11%	11%	
	College/university	69%	68%	
	Postgraduate	20%	21%	n.s.
Father obesity	Normal weight	32%	45%	
	Overweight/obese	69%	55%	C>I
Mother obesity	Normal weight	99%	95%	
	Overweight/obese	1%	5%	n.s.
† Comparison based on F-test for continuous measures and chi-square tes for categorical measures ($\alpha \leq 0.05$)				
