# Supplementary material for: A policy-driven multifaceted approach for early childhood physical fitness promotion: impacts on body composition and physical fitness in young Chinese children
Source: BMC Pediatr. 2014 May 5;14:118. doi: 10.1186/1471-2431-14-118 (PMC4108008; doi:10.1186/1471-2431-14-118)
Supplement: Additional file 3 — Changes of reported exercise and fitness assessment in intervention parents and teachers from baseline to posttest. [file 1471-2431-14-118-S3.pdf]

Additional file 3 – Changes of reported exercise and fitness assessment in intervention parents and teachers from baseline to posttest

		Baseline (%)	Posttest (%)
Parent's reported frequency of exercise†±	No exercise	53.3	30.2
	Once a week	30.8	42.3
	Two or more times a week	15.9	27.6
Parent's reported duration of exercise sessions†±	Exercise <30 min/session	54.1	9.3
	Exercise 30-60 min/session	34.7	47.4
	Exercise >60 min/session	11.2	43.3
Parent levels of physical fitness assessment†*	Poor	6.3	1.7
	Fair	29.5	27.9
	Good	40.6	28.5
	Outstanding	23.6	41.9
Teacher levels of physical fitness assessment†*	Poor	10.7	0.0
	Fair	35.7	17.9
	Good	21.4	50.0
	Outstanding	32.1	32.1

† positive linear trend based on chi-square test, $\alpha \leq 0.01$
± based on self-reports of physical activities from the parental surveys
* based on physical fitness index scores of Chinese Adult Physical Fitness Test Standards[6]
